# Palmitic Acid-Induced Neuron Cell Cycle G_2_/M Arrest and Endoplasmic Reticular Stress through Protein Palmitoylation in SH-SY5Y Human Neuroblastoma Cells

**DOI:** 10.3390/ijms151120876

**Published:** 2014-11-13

**Authors:** Yung-Hsuan Hsiao, Ching-I Lin, Hsiang Liao, Yue-Hua Chen, Shyh-Hsiang Lin

**Affiliations:** 1 Department of School of Nutrition and Health Sciences, Taipei Medical University, 250 Wu-Hsing Street, Taipei 110, Taiwan; E-Mails: m507100009@tmu.edu.tw (Y.-H.H.); simonleo@seed.net.tw (H.L.); yuehwa@tmu.edu.tw (Y.-H.C.); 2 Department of Nutrition and Health Sciences, Kainan University, No.1 Kainan Road, Luzhu Shiang, Taoyuan 338, Taiwan; E-Mail: cilin@mail.knu.edu.tw

**Keywords:** obesity, neurodegenerative disease, saturated fatty acids, palmitic acid, cell cycle arrest, endoplasmic reticular stress, neurons, protein palmitoylation

## Abstract

Obesity-related neurodegenerative diseases are associated with elevated saturated fatty acids (SFAs) in the brain. An increase in SFAs, especially palmitic acid (PA), triggers neuron cell apoptosis, causing cognitive function to deteriorate. In the present study, we focused on the specific mechanism by which PA triggers SH-SY5Y neuron cell apoptosis. We found that PA induces significant neuron cell cycle arrest in the G_2_/M phase in SH-SY5Y cells. Our data further showed that G_2_/M arrest is involved in elevation of endoplasmic reticular (ER) stress according to an increase in p-eukaryotic translation inhibition factor 2α, an ER stress marker. Chronic exposure to PA also accelerates beta-amyloid accumulation, a pathological characteristic of Alzheimer’s disease. Interestingly, SFA-induced ER stress, G_2_/M arrest and cell apoptosis were reversed by treatment with 2-bromopalmitate, a protein palmitoylation inhibitor. These findings suggest that protein palmitoylation plays a crucial role in SFA-induced neuron cell cycle G_2_/M arrest, ER stress and apoptosis; this provides a novel strategy for preventing SFA-induced neuron cell dysfunction.

## 1. Introduction

Alzheimer’s disease (AD) is the most common neurodegenerative disease [1], which is characterized by cognitive decline, memory dysfunction and behavioral impairments [2]. Epidemiological studies indicate that a high saturated fat dietary pattern is associated with cognitive decline and neurodegenerative diseases (NDs) [3,4,5]. Clinical studies also showed that people in the upper fifth of saturated fatty acid (FA; SFA) intake had 2.2-times the risk of AD incidence compared to people in the lowest fifth [6]. Furthermore, brains and cerebrospinal fluid (CSF) of AD patients are also characterized by elevated SFAs compared to healthy controls [7,8]. Therefore, increased SFAs in the brain and CSF may contribute to AD pathology [8]. Pathological changes in AD include beta-amyloid accumulation and beta-secretase activation, which play important roles in controlling synaptic activity and triggering neuronal apoptosis [9].

Recent studies showed that palmitic acid (PA), a 16:0 SFA, is significantly elevated in brains of AD patients [7]. PA is the most common saturated long-chain FA in food [10] and is rich in plant and animal products, like palm oil, coconut oil, cheese, butter and lard [11]. According to animal and human studies, diets high in PA induce cognitive decline [12,13,14]; recent *in vitro* studies also showed that PA treatment induces neuronal cell apoptosis [15,16], which suggests that PA may accelerate the pathologies of NDs [17,18,19]. There are two sources of brain PA: dietary PA and *de novo* lipogenesis [20]. Dietary PA can be transported through the vascular system and pass through the blood-brain-barrier [21]. It was found that both PA and oleic acid (OA) are elevated in AD patients’ brains [7]. Further studies then examined whether PA and OA elevation affected AD development, and the results indicated that PA induction caused AD-like pathological changes and cell apoptosis in primary cortical neurons, whereas OA treatment did not induce such changes or neuronal cell death [22,23,24].

A connection between AD and type 2 diabetes (T2D) was suggested [25,26]. Since observational studies showed that patients with T2D have higher risks of AD development, researchers begun revealing pathogenic pathways shared by AD and T2D [27,28]. One of the shared characteristics of AD and T2D is hyperglycemia. It was found that subjects with impaired glycemic control have higher cognitive decline than subjects with normal glycemic control [29]. Moreover, high-fat diet-induced hyperglycemia was correlated with the development of AD pathology in rodents [30]. Hyperglycemia induces the elevation of PA through lipogenesis [31,32], which is also one of the main sources of elevated blood SFA levels. In addition, T2D patients with poor glycemic control exhibited higher levels of SFAs in the blood and lower cognitive function [33,34,35]. SFAs, especially PA, induced neurotoxicity in cell culture. Therefore, deterioration of memory function in T2D patients may be associated with elevated blood SFAs [24,36,37]. Studies indicated that the longer patients suffer from diabetes, the greater chances they have of developing AD [38,39,40,41].

Cell cycle regulation is an essential process of cell growth, differentiation and proliferation in neurons [42]. Dysregulation of the cell cycle causes neuronal cell dysfunction and cell death [43,44]. Studies also demonstrated that PA treatment is related to the disruption of the cell cycle in pancreatic beta-cell and hepatic cells [45,46,47]. In addition, studies also supported a role of endoplasmic reticular (ER) stress in SFA-induced cell death [45]. In other aspects, dysregulation of protein palmitoylation was suggested as participating in PA-induced ER stress and beta-cell toxicity [48]. Palmitoylation is a process of adding the 16-carbon SFA, palmitate, via thioester linkage to a cysteine residue, which regulates neuronal protein trafficking and function [49]. The purpose of this study was to investigate whether the SFA, PA, induces neuronal toxicity via disturbing the cell cycle and the role of palmitoylation in PA-induced ER stress in SH-SY5Y human neuroblastoma cells.

## 2. Results and Discussion

### 2.1. Incorporation of FAs into SH-SY5Y Cells

**Figure 1:**
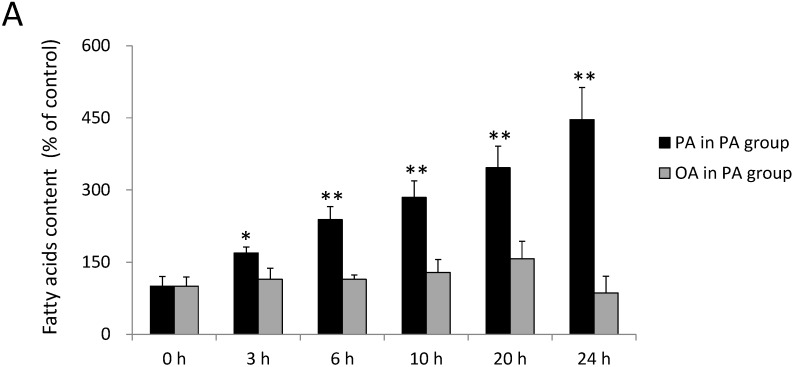

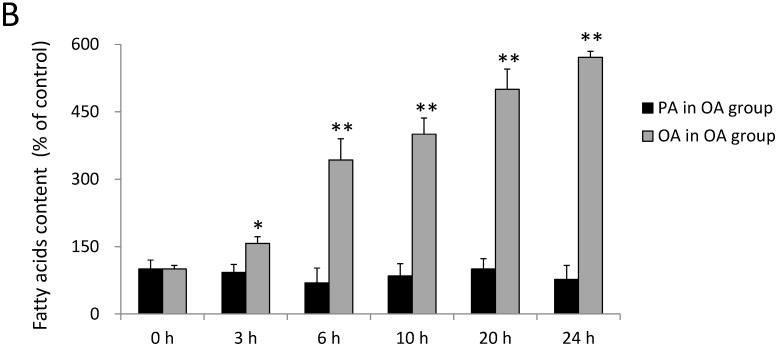
Relative fatty acid contents of SH-SY5Y cells treated with palmitic acid (PA) or oleic acid (OA) at 0, 3, 6, 10, 20 and 24 h. The fatty acid composition was analyzed by gas chromatography after 0~24 h of incubation with (**A**) 0.3 mM PA and (**B**) 0.3 mM OA. Data were analyzed by a one-way analysis of variance (ANOVA), followed by the LSD test, and are presented as the mean ± SD. * *p* < 0.05, ** *p* < 0.01 *vs.* the control group (*n* = 3).

FA uptake was analyzed by gas chromatography. As shown in Figure 1A, the PA content significantly increased in cells compared to the control group after 3, 6, 10, 20 and 24 h of incubation with 0.3 mM PA (*p* < 0.05). The PA content increased by 69% ± 12%, 138% ± 27%, 185% ± 34%, 246% ± 45% and 346% ± 67%, respectively, in SH-SY5Y after 3, 6, 10, 20 and 24 h of incubation with 0.3 mM PA (Figure 1A). On the other hand, the OA content also increased by 57% ± 12%, 243% ± 27%, 300% ± 34%, 400% ± 45% and 471% ± 67%, respectively, after 3, 6, 10, 20 and 24 h of incubation with 0.3 mM OA (Figure 1B). These data suggested that FA uptake was elevated by FA treatment in SH-SY5Y cells. The treated FAs, including PA and OA, were incorporated into SH-SY5Y cells from the medium and reached significant levels after 3 h of incubation.

### 2.2. Effects of PA and OA on SH-SY5Y Cell Viability

**Figure 2:**
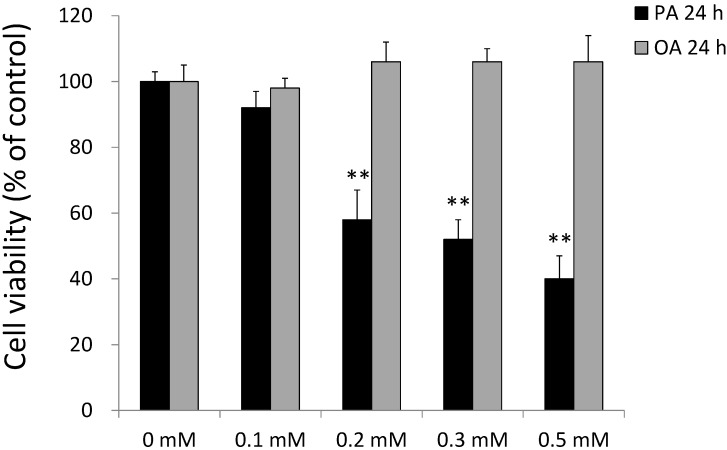
The effects of palmitic acid (PA) and oleic acid (OA) on cell viability of SH-SY5Y cells. SH-SY5Y cells were treated with the vehicle control (0 mM), 0.2~0.5 mM PA, or 0.2~0.5 mM OA for 24 and 48 h. After treatment, cell viability was measured by an MTT assay. All treatment conditions were normalized to the control group. Data were analyzed by a one-way ANOVA, followed by the LSD test, and are presented as the mean ± SD. ** *p* < 0.01 *vs.* the control group.

The effects of PA and OA on the viability of SH-SY5Y cells were evaluated using an MTT assay. As shown in Figure 2, treatment with 0.2~0.5 mM PA resulted in a significant decrease in cell viability after incubation for 24 and 48 h. On the contrary, OA treatment at 0.2~0.5 mM for 24 and 48 h did not alter cell viability. PA significantly impaired the viability of SH-SY5Y cells in dose- and time-dependent manners at concentrations of 0.2, 0.3 and 0.5 mM for 24 and 48 h (*p* < 0.05) (Figure 2). Compared to results at 24 h, the viability of SH-SY5Y cells had significantly decreased at 48 h at doses of PA of 0.3 and 0.5 mM (*p* < 0.05) (Figure 2).

### 2.3. Effects of PA on SH-SY5Y Cell Morphology

**Figure 3:**
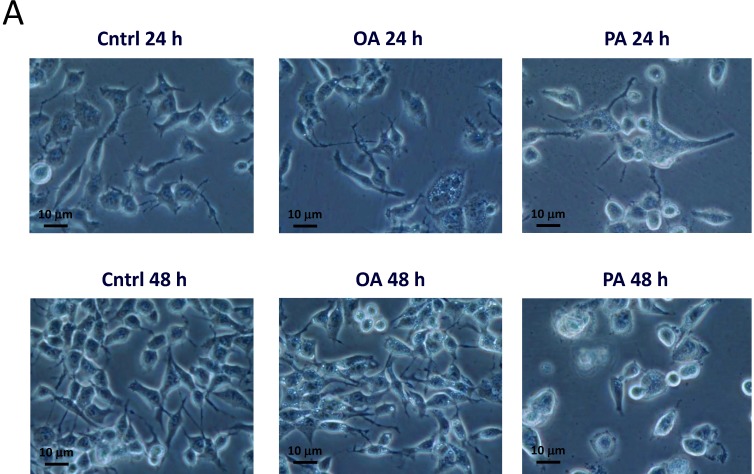

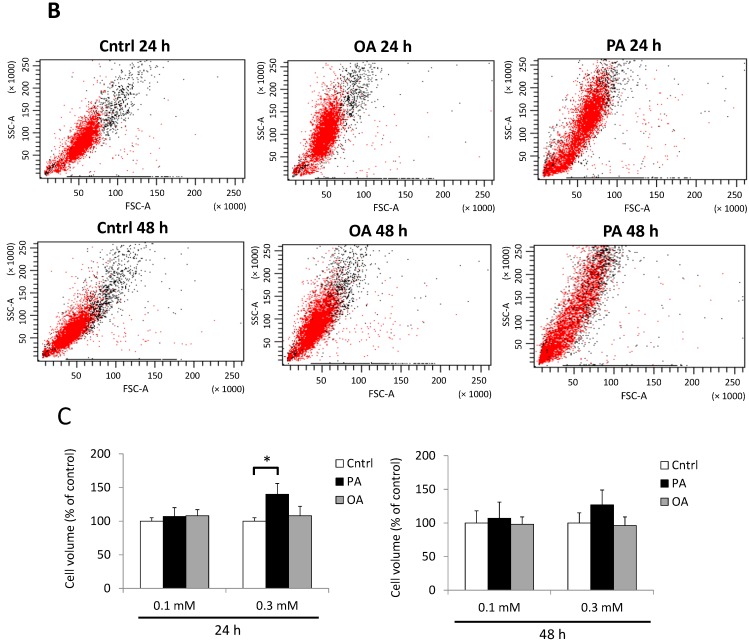
Effects of palmitic acid (PA) and oleic acid (OA) on SH-SY5Y cell morphology. (**A**) Cells were treated with the vehicle control (Cntrl), 0.3 mM OA or 0.3 mM PA for 24 (**top**) and 48 h (**bottom**) and then imaged with a microscope; (**B**) effects of PA and OA on the SH-SY5Y cell size. Cells were treated with the vehicle (Cntrl), 0.3 mM OA or 0.3 mM PA for 24 (**top**) and 48 h (**bottom**), then measured with flow cytometry; (**C**) Effects of PA and OA on the SH-SY5Y cell volume. Cells were treated with PA or OA for 24 and 48 h, and then, the cell volume was quantified by flow cytometry. Data were analyzed by a one-way ANOVA, followed by the LSD test, and are presented as the mean ± SD. * *p* < 0.05 *vs.* the Cntrl (*n* = 3).

We next observed morphological alterations of SH-SY5Y cells treated with different concentrations of PA and OA for 24 and 48 h. As shown in Figure 3, SH-SY5Y cells exhibited obvious morphological changes after treatment with 0.3 mM PA for 24 h (Figure 3A). The morphology of the PA-treated group showed larger cell sizes and fading synapses, and cells were unattached to the culture dish (Figure 3A). In order to confirm the visual observations, we further measured the cell size using flow cytometry (Figure 3B,C). We found that the cell size was significantly larger in the group treated with 0.3 mM PA after 24 h of incubation, but not after 48 h of incubation (Figure 3C).

On the other hand, OA treatment did not alter the morphology of SH-SY5Y cells. After incubation with 0.3 mM OA, the OA-treated group showed no differences in morphology compared to the control. Furthermore, we also evaluated the cell volume by flow cytometry to confirm our visual findings. The mean cell volume (Figure 3C) of the PA-treated group was significantly elevated to 140% ± 16% at 24 h (*p* < 0.05). Groups treated with OA exhibited no significant difference compared to the control.

### 2.4. Cell Cycle G_2_/M Arrest Induced by PA in SH-SY5Y Cells

**Figure 4:**
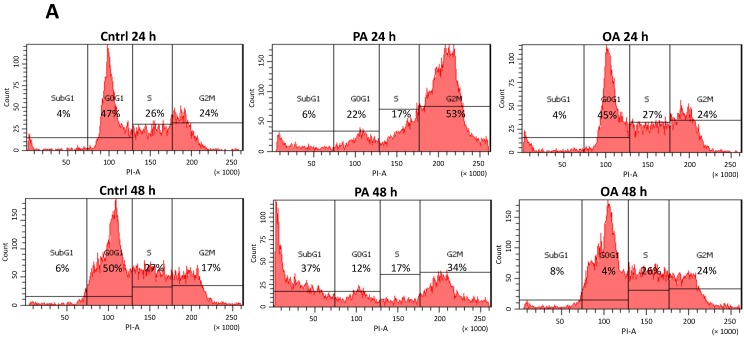

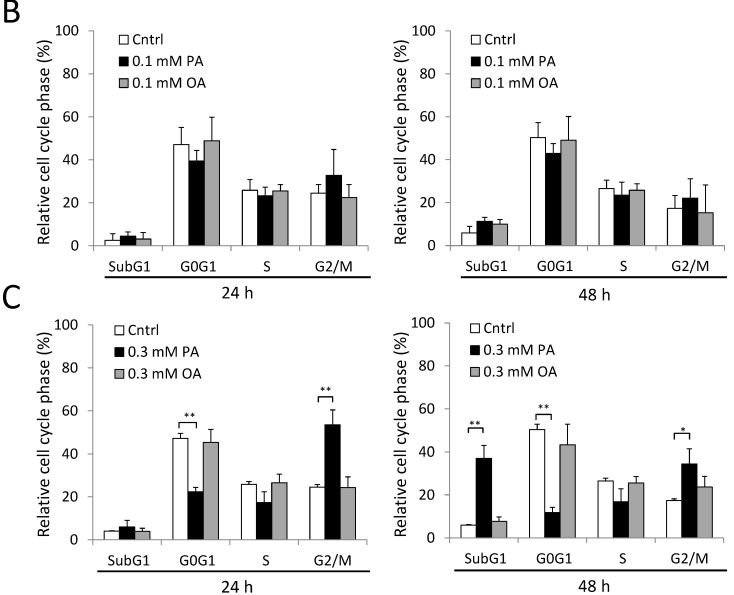
Effects of palmitic acid (PA) and oleic acid (OA) on the SH-SY5Y cell cycle. (**A**) Cells were treated with the vehicle control (Cntrl), 0.3 mM PA or 0.3 mM OA for 24 (**top**) and 48 h (**bottom**). The cell cycle was then analyzed by flow cytometry; (**B**) Quantified data are shown as the percentages of the four phases divided by the DNA content in the cell cycle. SH-SY5Y cells were treated with the vehicle control (Cntrl), 0.1 mM PA or 0.1 mM OA for 24 (**top**) and 48 h (**bottom**) and (**C**) Cells were treated with the vehicle control (Cntrl), 0.3 mM PA or 0.3 mM OA for 24 (**top**) and 48 h (**bottom**). Data were analyzed by a one-way ANOVA, followed by the LSD test, and are presented as the mean ± SD. * *p* < 0.05 *vs* the Cntrl group. ** *p* < 0.01 *vs* the Cntrl group (*n* = 3).

In the cell cycle examination, we found that PA dramatically induced cell cycle arrest at the G_2_/M phase after treatment with 0.3 mM PA for 24 h (Figure 4). Fluorescence integration of the G_2_/M phase significantly increased (*p* < 0.01), while the G_0_G_1_ phase significantly decreased (*p* < 0.05) after treatment with 0.3 mM PA for 24 h (Figure 4C), indicating that cells had lower rates of growth and tended to be arrested at the G_2_/M transition. In addition, the 0.1-mM PA-treated group did not exhibit significant G_2_/M arrest, which suggests that the lower dose of PA had minor effects on the neuronal cell cycle (Figure 4B). On the contrary, OA treatment did not disturb the cell cycle; neither the G_0_G_1_ phase nor G_2_/M phase differed from the control group after 24 and 48 h of incubation with OA (Figure 4B,C). In addition to G_2_/M arrest, we further observed increased apoptotic cells, indicated by an increase in the subG_1_ phase, after treatment with 0.3 mM PA for 48 h by flow cytometry (Figure 4C). While significant cell apoptosis was observed after treatment with 0.3 mM PA for 48 h, SH-SY5Y cells showed reduced growth as indicated by reduced rates of the G_0_/G_1_ phase. Based on the above results, these observations provide evidence that PA and OA have distinct effects on the SH-SY5Y cell cycle and indicate that chronic exposure of SH-SY5Y to PA evoked significant G_2_/M arrest prior to neuronal apoptosis. We are the first group to observe neuronal cell cycle G_2_/M arrest evoked by the SFA, PA, and so, we then worked to find the possible cause of the G_2_/M arrest induced by PA.

### 2.5. ER Stress and Beta-Amyloid Accumulation Induced by PA

**Figure 5:**
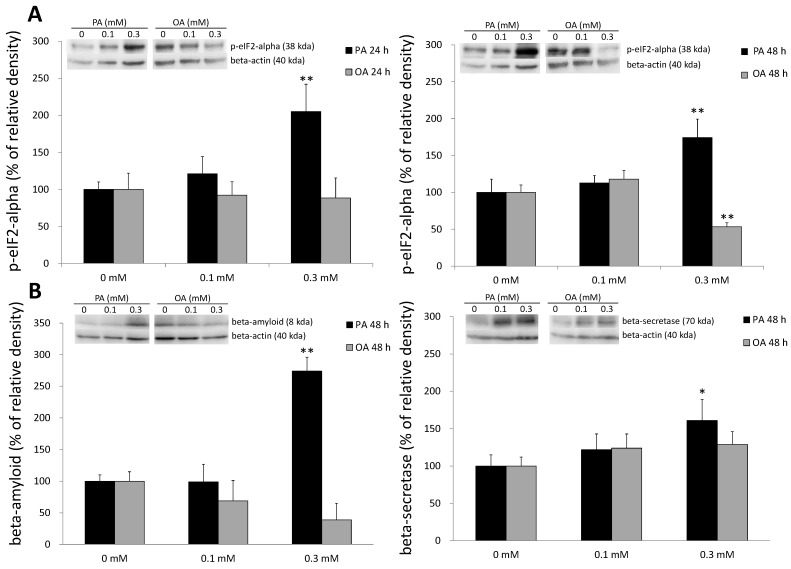
Effects of palmitic acid (PA) and oleic acid (OA) on Alzheimer’s disease pathology-related protein expression and an endoplasmic reticular (ER) stress marker. (**A**) SH-SY5Y cells were treated with 0.1~0.3 mM PA or OA for 24 (**left**) or 48 h (**right**). Cells were then harvested for the analysis of protein expression of the ER stress marker, p-eukaryotic initiation factor 2-alpha (p-eIF2α) and (**B**) SH-SY5Y cells were incubated with PA or OA for 48 h. Cells were then harvested for examination of beta-amyloid protein expression (**left**). SH-SY5Y cells were incubated with PA or OA for 48 h, and then, the beta-secretase protein expression was examined (**right**). Data were analyzed by a one-way ANOVA, followed by the LSD test, and are presented as the mean ± SD. * *p* < 0.05 *vs* the control (0 mM PA; 0 mM OA). ** *p* < 0.01 *vs* the control (0 mM PA; 0 mM OA) (*n* = 3).

ER stress was suggested to play an important role in SFA-induced lipotoxicity [50,51]. To further examine the effects of PA on ER stress, we analyzed the protein expression of p-eIF2a, an ER stress marker, in PA-treated cells. We found that ER stress significantly increased in the PA-treated group (Figure 5A), which indicates that G_2_/M arrest in neuronal cells might be a result of PA-induced ER stress. Furthermore, PA also stimulated beta-secretase protein expression and beta-amyloid accumulation in SH-SY5Y cells (Figure 5B), especially after 48 h of PA treatment. These data demonstrated that PA-induced neuronal apoptosis was similar to that in a cell model of AD. On the contrary, OA induction did not induce beta-secretase protein expression or beta-amyloid accumulation in any of the OA-treated groups.

### 2.6. Antioxidants and a PKC Inhibitor could not Alleviate PA-Induced Cell Death

**Figure 6:**
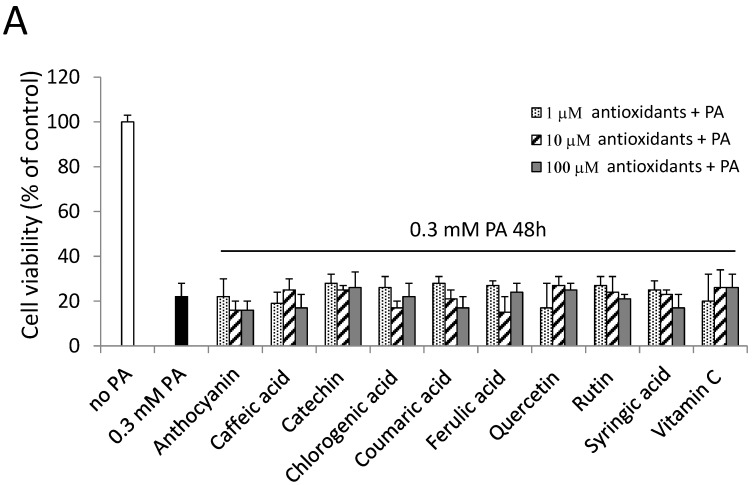

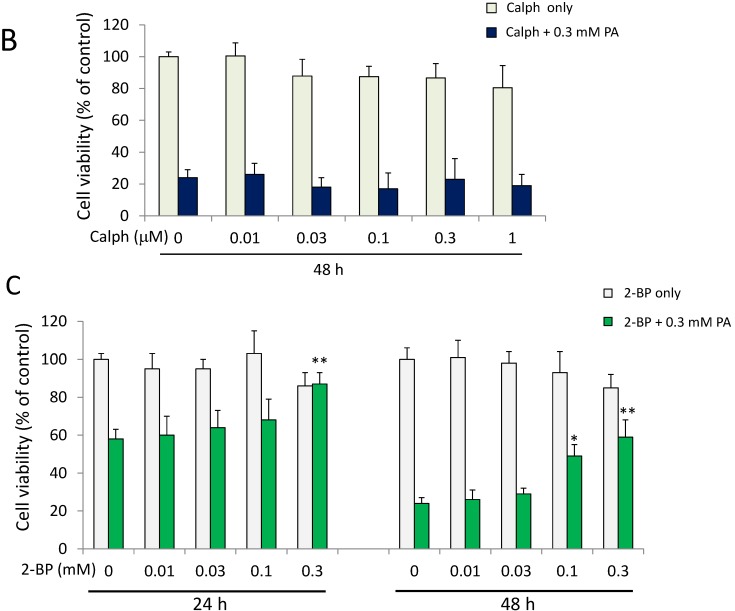
Effects of antioxidants, a protein kinase C (PKC) inhibitor (calphostin C; Calph) and a palmitoylation inhibitor (2-bromopalmitate; 2-BP) on PA-induced cell death. (**A**) SH-SY5Y cells were treated with antioxidants, including polyphenols and vitamin C, 1 h before treatment with 0.3 mM PA. The cell viability of different groups was normalized to the control group (no PA); (**B**) SH-SY5Y cells were treated with the PKC inhibitor, Calph, for 1 h before treatment with 0.3 mM PA and light activation. The final concentrations of Calph were 0.01~1 μM. Cell viability of SH-SY5Y cells was normalized to the control (0 μM Calph); (**C**) Cells were treated with the palmitoylation inhibitor, 2-BP, 1 h before treatment with 0.3 mM PA and then were incubated for 24 and 48 h. Cell viability was normalized to the control (0 mM 2-BP without PA treatment). Data were analyzed by a one-way ANOVA, followed by the LSD test, and are presented as the mean ± SD. * *p* < 0.05, ** *p* < 0.01 *vs* the control group (*n* = 3).

Our results suggested that PA induced G_2_/M arrest through the induction of ER stress. It was shown that oxidative stress is involved in cell cycle G_2_/M arrest and ER stress induction [52]. Since phenolic acids and vitamin C are known for their antioxidative abilities, they were used to observe if PA-induced cell death could be alleviated through antioxidants in our study. However, pretreatment with antioxidants 1 h before treatment with 0.3 mM PA did not reverse PA-induced cell death in our study (Figure 6A), which suggested that antioxidation might not play a key role in the cytotoxicity induced by PA in SH-SY5Y cells. Cell viability showed no difference between the group with and that without antioxidants under PA treatment.

PKC was also suggested to be involved in cell cycle G_2_/M arrest and ER stress [53,54]. Calphostin C, a broad PKC inhibitor [55], was used in this study. However, calphostin C pretreatment did not reverse PA-induced cell death (Figure 6B), which indicates that PA-induced cell death did not mainly occur through PKC activation. Pretreatment with either a PKC inhibitor or antioxidants did not alleviate PA-induced cell death. Nevertheless, we found that pretreatment with 2-BP, a protein palmitoylation inhibitor, reversed cell viability after 24 and 48 h of treatment with 0.3 mM PA in SH-SY5Y cells (Figure 6C). Moreover, the beneficial actions of 2-BP were concentration related, with 0.3 mM 2-BP providing maximal protection after treatment with PA for 24 and 48 h (Figure 6C).

### 2.7. A Protein Palmitoylation Inhibitor Attenuated PA-Induced G_2_/M Arrest and ER Stress

**Figure 7:**
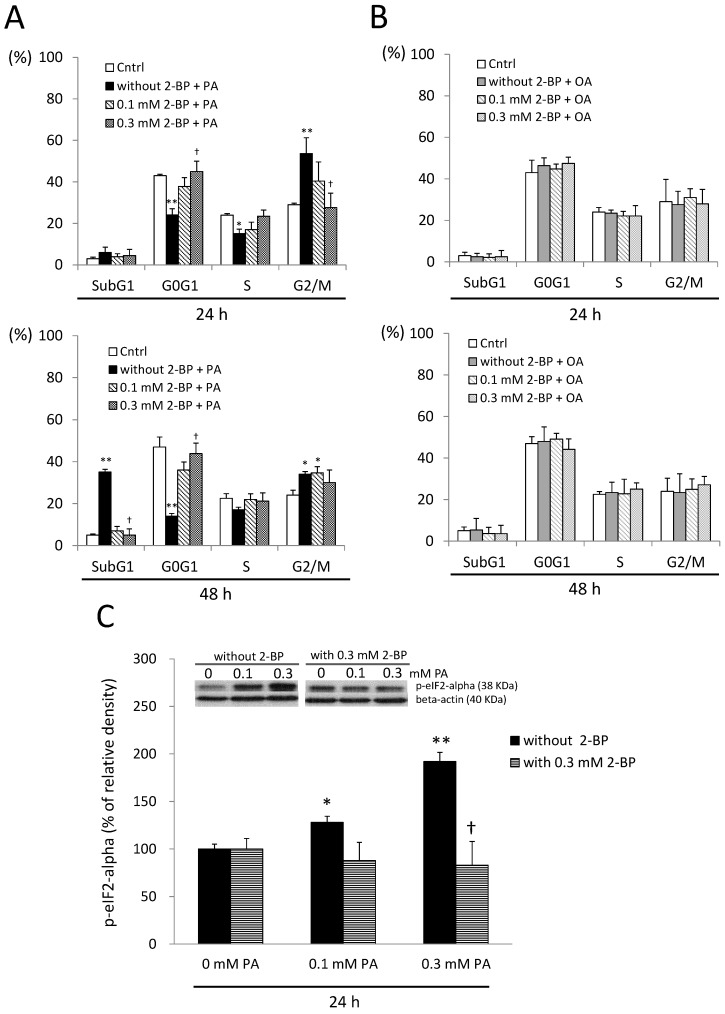
Effects of a protein palmitoylation inhibitor on palmitic acid (PA)-induced cell cycle disturbance and endoplasmic reticular (ER) stress. (**A**) SH-SY5Y cells were treated with the vehicle (Cntrl) and 0.3 mM PA with or without 2-bromopalmitate (2-BP) for 24 and 48 h, and then, the cell cycle was examined by flow cytometry; (**B**) SH-SY5Y cells were treated with the vehicle (Cntrl) and 0.3 mM OA with or without 2-BP for 24 and 48 h, and the cell cycle was examined by flow cytometry; (**C**) effects of a protein palmitoylation inhibitor on ER stress in PA-treated SH-SY5Y cells at 24 h. Data were analyzed by a one-way ANOVA, followed by the LSD test, and are presented as the mean ± SD. * *p* < 0.05, ** *p* < 0.01 *vs* the Cntrl; ^†^ *p* < 0.05 *vs* groups with PA without 2-BP. (*n* = 3).

Since 2-BP was effective at preventing cell death in response to PA, the effects of 2-BP on the cell cycle were also examined. As a result, 2-BP significantly attenuated G_2_/M arrest induced by PA (*p* < 0.05) (Figure 7A), whereas OA treatment did not (Figure 7B). In addition, pretreatment with 2-BP mitigated ER stress induced by PA (*p* < 0.05) (Figure 7C). These findings suggest that aberrant protein palmitoylation may contribute to PA toxicity toward neuronal cells. When examining other possible pathways by which PA activates ER stress and G_2_/M arrest, we found that treatment with the protein palmitoylation inhibitor, 2-BP, prevented PA-mediated ER stress induction and G_2_/M arrest, indicating that protein palmitoylation is pivotal to this process.

### 2.8. Discussion

The present study elucidates the effects of PA on SH-SY5Y cell apoptosis and includes three main findings. Firstly, we demonstrated that PA caused conspicuous morphological changes in SH-SY5Y human neuroblastoma cells. A relatively larger cell volume was observed after exposure to PA. Secondly, we found that the changes in the volume of cells occurred along with the arrest of the cell cycle at the G_2_/M phase (Figure 3 and Figure 7). We provide the first direct evidence that PA induces G_2_/M arrest through protein palmitoylation. Thirdly, we characterized the importance of protein palmitoylation in PA-induced ER stress, G_2_/M arrest and neuronal cell apoptosis. Together, these findings contribute to the understanding of the mechanism(s) in which PA decreases neuronal cell survival.

Studies have shown that elevated SFA levels are associated with the pathological development of neurological disorders [13,56,57,58]. For instance, a high-SFA diet and an elevation of plasma SFA were proposed to be risk factors for NDs, especially AD [6,59]. SFAs, such as PA and stearic acid (SA), can cause deleterious effects in many cell types [15,24,60,61,62,63], with apoptosis being the most common phenomenon [63,64]. Therefore, it is critical to investigate possible mechanisms of PA-induced neuronal apoptosis. In this study, we selected PA as a representative SFA to explore its effects on neuronal SH-SY5Y cells, while OA was used as a reference. Though OA was indicated to have different effects at certain concentrations (0.1 and 0.3 mM) under the diabetic cell model [65], we did not find any adverse effect in our study.

In the present study, we found that the fluorescent intensity of the G_2_/M phase substantially increased, which indicates a growth/mitosis-arrested status in SH-SY5Y cells after 24 h of PA incubation. Recent studies also showed that PA induced apoptosis in neuronal cells [15,23,66,67]. Herein, we showed that direct G_2_/M arrest occurs in neuronal cells exposed to PA. It was proposed that neuronal apoptosis detected in NDs, such as AD, was related to the blockade of the cell cycle at the G_2_/M phase [43]. However, in acute central nervous system (CNS) injury, such as stroke and trauma, the cell cycle is arrested at the G_1_/S transition, which indicates that the adverse alterations induced by PA are similar to NDs, rather than acute injury to neuronal cells [43]. Since the G_2_/M arrest occurs prior to apoptosis [68,69,70], we inferred that G_2_/M arrest led to neuronal apoptosis in PA-treated SH-SY5Y cells, which results in a decrement in cell size. Sustained elevation of PA in the brain is known to cause deterioration of neuronal cell viability [71,72]. One key mechanism in SFA-mediated cell stress is ER stress [15,73]. A previous study also suggested that G_2_/M arrest can be induced by ER stress in non-neuronal cells [70]; therefore, we suggest that the G_2_/M arrest appearing in SH-SY5Y cells may be involved in ER stress. In agreement with previous findings, we found that PA was able to increase the ER stress marker, p-eIF2α, and promote cell death. We also demonstrated that PA accelerates AD-like pathological changes in SH-SY5Y cells, such as the accumulation of beta-amyloid (Figure 5A) and the elevation of beta-secretase. Although previous studies reported that PA, through its uptake and metabolism by astrocytes, led to AD-like pathological changes in neurons [71], our work demonstrated that PA can directly induce significant beta-amyloid accumulation and beta-secretase protein expression without stimulating astrocytes.

Oxidative stress is involved in cell-cycle G_2_/M arrest, ER stress induction and cell death [52]. However, in our findings, antioxidants, like phenolic acid and vitamin C, could not reverse the lowered cell viability induced by PA. These data suggest that oxidative stress may play a marginal role in PA-induced cell death. PKC was also suggested to be involved in the cell cycle G_2_/M checkpoint in a breast cancer cell line [53]. In addition, ER stress can be induced by the activation of the PKC isoforms, PKC-theta and PKC-delta [74,75]. One study showed that neurons with higher expression of PKC were critical for nutrient sensing and were responsive to PA, suggesting that PA treatment may also be related to PKC activation, thus stimulating neuronal cell apoptosis [76]. Another study also confirmed that inhibiting PKC activation downregulated cell apoptosis [77]. Nevertheless, in the present study, the PKC inhibitor, calphostin C, could not reverse cell death induced by PA.

Protein palmitoylation is a post-translational process in which FAs, primarily PA, are bound to cysteine residues through thio-ester linkages [78]. Protein palmitoylation regulates diverse aspects of neuronal protein trafficking and functions [49]. A global study also identified 68 known neuronal palmitoylated proteins and 200 new candidates, including neurotransmitters, receptors and transporters, indicating that palmitoylation plays an extremely important role in regulating neuronal functions [49]. Attachment of PA alters a protein’s structure, hydrophobicity and translocation [79]. Protein palmitoylation is also highly dynamic [80] and regulates protein localization and functions in neuron synapses [81]. Furthermore, proteins with cysteine residues can be palmitoylated *in vitro* in the presence of FAs [78,82]. The spontaneous process of this modification suggests that protein palmitoylation may increase in the presence of elevated PA.

In the present study, we found that PA stimulated ER stress via protein palmitoylation in SH-SY5Y cells. Since ER is sensitive to aberrant proteins, uncontrolled protein palmitoylation could exacerbate ER stress [83,84]. Previous studies associated ER stress with lipotoxicity [73,85], and unregulated protein palmitoylation could be a potent activator of protein aggregation and ER stress [84]. In our study, we applied 2-BP to examine the inhibitory effects of protein palmitoylation. 2-BP, a non-metabolized PA analog, blocks PA incorporation into proteins [86,87,88].

In our results, 2-BP significantly attenuated the toxicity of PA toward SH-SY5Y cells. The effects of 2-BP on PA-induced ER stress were also examined. We also found that the PA-induced ER stress marker, p-eIF2α, was attenuated by treatment with 2-BP in SH-SY5Y cells. Since prolonged ER stress can culminate in G_2_/M blockage, we examined the effect of 2-BP on PA-induced G_2_/M arrest. Consistent with the attenuation of ER stress, the presence of 2-BP prevented PA-induced G_2_/M arrest. In conclusion, we provide evidence that suggests that the SFA, PA, induces deleterious effects on neuronal cells through protein palmitoylation. Taken together, these findings suggest that PA induces neuronal ER stress, G_2_/M arrest and cell apoptosis through protein palmitoylation.

## 3. Experimental Section

### 3.1. Cell Culture Preparation

The SH-SY5Y human neuroblastoma cell line, a well-established cell model for studying ND diseases [89], was purchased from American Type Culture Collection (ATCC, Manassas, VA, USA). Cells were cultured in minimum essential medium (MEM) supplemented with F12 (Gibco, Paisley, UK), 10% fetal bovine serum, 1% non-essential amino acids (NEAAs), sodium bicarbonate and 1% penicillin-streptomycin (pen/strep) at 37 °C in a 5% CO_2_ incubator. Fresh medium was changed every 2~3 days. Each aliquot (vial) of cells was passed through no more than 10 passages. Experiments were performed at 50% cell confluence.

### 3.2. FA Treatment

PA and OA were purchased from Sigma-Aldrich (Dorset, UK). The FA/bovine serum albumin (BSA) complex was prepared by a modified method [62]. Briefly, 20 mM PA and OA stock solutions were prepared in 0.01 N NaOH by heating to 80 °C in a dry bath. Stocks were then added to MEM containing a 1% BSA solution to reach final concentrations of 0.1~0.5 mM. Solutions were next vortex-mixed followed by a further incubation for 30 min at 37 °C. The PA and OA complex solutions were then immediately used to treat SH-SY5Y cells for 24 or 48 h according to the experimental design.

### 3.3. Cell Viability Assay

A 3-[4,5-cimethylthiazol-2-yl]-2,5-diphenyl tetrazolium bromide (MTT) assay was performed to determine the viability of SH-SY5Y cells under the influence of PA and OA [90]. Briefly, 24 or 48 h following treatment with PA and OA, MTT was added to each well of a 24-well plate and incubated at 37 °C for 1 h. Purple-colored precipitates of the living cell metabolite, formazan, were then dissolved in 500 µL of dimethyl sulfoxide (DMSO) and were analyzed in 96-well plates. The color absorbance was recorded at 590 nm. Cell viability was calculated by the absorbance ratio of the treatment group over the control group.

### 3.4. Cell Size Measurement

The cell size of SH-SY5Y is determined by the intensity of forward light scattering (FSC), the extent of the size of the scattering cells, by flow cytometry. Briefly, SH-SY5Y cells were stained with propidium iodide (PI) solution and incubated in the dark for 30 min at 37 °C, then excited at 488 nm and measuring the emission at 580 nm in a flow cytometer. FSC for viable cells was generated on a computer from raw data files of flow cytometry.

### 3.5. FA Composition Analysis

To determine FA uptake by SH-SY5Y cells, total FAs were extracted by the widely used method of Folch *et al.* [91]. Briefly, FAs were extracted with chloroform and methanol, and 0.02% CaCl_2_ was added to stabilize the phospholipids. Lysates were then vortex-mixed and centrifuged. A mixture of chloroform:methanol:water (3:48:47; 0.5 mL) was added to the supernatant. After further vortex-mixing and centrifugation, FAs extracted from the chloroform layer were dried in a vacuum evaporator. FAs were then transmethylated with 0.15 mL of 14% boron fluoride in methanol at 100 °C for 10 min [92]. Then, samples were cooled on ice, pentane and water added and the two phases separated by centrifugation. The pentane phase was transferred to a new tube and dried in a vacuum evaporator. The methyl esters were dissolved in hexane and analyzed by Thermo Scientific Focus gas chromatography (GC) (Thermo Scientific, Courtaboeuf, France) [93].

### 3.6. Cell Cycle Analysis

The cell cycle was examined by a modified flow cytometric method [94]. In brief, SH-SY5Y cells were harvested at 24 and 48 h. Harvested cells were washed twice with phosphate-buffered saline (PBS) and fixed in ice-cold 70% ethanol at 4 °C overnight. Ethanol-fixed cells were then stored at 4 °C for up to 1 month. Ethanol-fixed cells were centrifuged and washed once with PBS. The cell pellet was then suspended in 0.2 mL DNA extraction buffer (192 mL 0.2 M Na_2_HPO_4_ and 8 mL 0.1 M citric acid, pH 7.8) and incubated in a 37 °C shaker for 30 min. After incubation, cells were centrifuged at 1500 rpm for 10 min. The supernatant was carefully discarded. Then, the pellet was stained with a propidium iodide (PI) solution (0.08 mg PI, 0.1% triton X-100 and 0.1 mg/mL RNase A in PBS) and incubated in the dark for 30 min at 37 °C. In total, 10,000 cells (adherent and non-adherent) were harvested. Cellular DNA contents were measured by exciting PI at 488 nm and measuring the emission at 580 nm using a BD canto II flow cytometer (BD Biosciences, San Jose, CA, USA).

### 3.7. Immunoblotting

To determine protein expressions by SH-SY5Y cells, cells were washed twice with PBS and solubilized in lysis buffer containing freshly added protease and phosphatase inhibitors. Protein aliquots were solubilized in Laemmli buffer and boiled at 95 °C for 5 min, and then, 10%~15% sodium dodecyl sulfate polyacrylamide gel electrophoresis (SDS-PAGE) was run. After electrophoresis, proteins were transferred to polyvinylidene difluoride (PVDF) membranes. The membranes were then blocked, washed and incubated with the antibody. Primary antibodies of p-eukaryotic translation inhibition factor 2α (p-eIF2α, 1:2000), beta-amyloid (1:2000) and beta-secretase (1:2000) were purchased from Cell Signaling Technology (Beverly, MA, USA). Target proteins were visualized using an enhanced chemiluminescence (ECL) system. Levels of beta-amyloid and beta-secretase were assessed by a densitometric analysis and normalized to the β-actin value.

### 3.8. Pretreatment with Antioxidants

In order to determine whether antioxidants are involved in PA-induced lipotoxicity, SH-SY5Y cells were incubated with 1~100 μM of antioxidants, including anthocyanin, caffeic acid, catechin, chlorogenic acid, coumaric acid, ferulic acid, rutin, syringic acid, vitamin C and quercetin. The antioxidants were dissolved in either DMSO or PBS according to their solubility, and cells were treated 1 h prior to FA treatment. Anthocyanin was extracted from Oriental plums; the extraction method was adopted from our previous protocol [95]. Caffeic acid, catechin, chlorogenic acid, coumaric acid, ferulic acid, rutin, syringic acid and vitamin C were purchased from Sigma-Aldrich (St. Louis, MO, USA), and quercetin was purchased from Wako Pure Chemical Industries (Osaka, Japan).

### 3.9. PKC Inhibitor Treatment

Calphostin C, a specific inhibitor of PKC [96], was purchased from Enzo Life Sciences (BML-EI-198-0100, Lausen, Switzerland). Since inhibition of PKC by calphostin C is light dependent and ordinary fluorescent light is sufficient for full activation [97], we exposed cells and medium containing 0.01~1 µM calphostin C to ordinary fluorescent light for 1 h at room temperature. SH-SY5Y cells were then treated with FAs and incubated at 37 °C in a 5% CO_2_ incubator.

### 3.10. Palmitoylation Inhibitor Treatment

2-Bromopalmitate (2-BP), a widely used palmitoylation inhibitor [81], is available from Sigma-Aldrich. A 100-mM stock was prepared in dimethyl sulfoxide (DMSO) and then diluted with media to final concentrations of 0.01~1 mM. SH-SY5Y cells were then incubated with 0.01~1 mM 2-BP to test its toxicity toward SH-SY5Y cells, and concentrations of 0.01~0.3 mM were selected for the following study. During the experiment, cells were treated with 0.01~0.3 mM 2-BP 1 h prior to FA treatment.

### 3.11. Statistical Analysis

Statistical analyses were performed using a one-way analysis of variance (ANOVA) followed by the least significant difference (LSD) test. A *p*-value of <0.05 was considered statistically significant.

## 4. Conclusions

Palmitic acid induced neuronal cell apoptosis, cell cycle G_2_/M arrest, beta-amyloid accumulation and the elevation of endothelial reticulum stress in SH-SY5Y human neuroblastoma cells. By inhibiting protein palmitoylation, the palmitic acid-induced cell apoptosis, cell cycle arrest and endothelial reticulum stress were significantly alleviated. These results suggest that protein palmitoylation plays an important role in palmitic acid-induced neurotoxicity.

## References

[1] Goedert M., Spillantini M.G. (2006). A century of Alzheimer’s disease. Science.

[2] LaFerla F.M. (2002). Calcium dyshomeostasis and intracellular signalling in Alzheimer’s disease. Nat. Rev. Neurosci..

[3] Solfrizzi V., D’Introno A., Colacicco A.M., Capurso C., Del Parigi A., Capurso S., Gadaleta A., Capurso A., Panza F. (2005). Dietary fatty acids intake: Possible role in cognitive decline and dementia. Exp. Gerontol..

[4] Kalmijn S. (1999). Fatty acid intake and the risk of dementia and cognitive decline: A review of clinical and epidemiological studies. J. Nutr. Health Aging.

[5] Kalmijn S., Launer L.J., Ott A., Witteman J., Hofman A., Breteler M. (1997). Dietary fat intake and the risk of incident dementia in the Rotterdam Study. Ann. Neurol..

[6] Morris M.C., Evans D.A., Bienias J.L., Tangney C.C., Bennett D.A., Aggarwal N., Schneider J., Wilson R.S. (2003). Dietary fats and the risk of incident Alzheimer disease. Arch. Neurol..

[7] Fraser T., Tayler H., Love S. (2010). Fatty acid composition of frontal, temporal and parietal neocortex in the normal human brain and in Alzheimer’s disease. Neurochem. Res..

[8] Fonteh A.N., Cipolla M., Chiang J., Arakaki X., Harrington M.G. (2014). Human Cerebrospinal Fluid Fatty Acid Levels Differ between Supernatant Fluid and Brain-Derived Nanoparticle Fractions, and Are Altered in Alzheimer’s Disease. PLoS One.

[9] Palop J.J., Mucke L. (2010). Amyloid-[beta]-induced neuronal dysfunction in Alzheimer’s disease: From synapses toward neural networks. Nat. Neurosci..

[10] Lovejoy J.C., Smith S.R., Champagne C.M., Most M.M., Lefevre M., DeLany J.P., Denkins Y.M., Rood J.C., Veldhuis J., Bray G.A. (2002). Effects of diets enriched in saturated (palmitic), monounsaturated (oleic), or trans (elaidic) fatty acids on insulin sensitivity and substrate oxidation in healthy adults. Diabetes Care.

[11] Gunstone F.D., Harwood J.L., Dijkstra A.J. (2010). The lipid handbook with CD-ROM.

[12] Morrison C.D., Pistell P.J., Ingram D.K., Johnson W.D., Liu Y., Fernandez-Kim S.O., White C.L., Purpera M.N., Uranga R.M., Bruce-Keller A.J. (2010). High fat diet increases hippocampal oxidative stress and cognitive impairment in aged mice: Implications for decreased Nrf2 signaling. J. Neurochem..

[13] Heude B., Ducimetière P., Berr C. (2003). Cognitive decline and fatty acid composition of erythrocyte membranes—The EVA Study. Am. J. Clin. Nutr..

[14] Pistell P.J., Morrison C.D., Gupta S., Knight A.G., Keller J.N., Ingram D.K., Bruce-Keller A.J. (2010). Cognitive impairment following high fat diet consumption is associated with brain inflammation. J. Neuroimmunol..

[15] Mayer C.M., Belsham D.D. (2010). Palmitate attenuates insulin signaling and induces endoplasmic reticulum stress and apoptosis in hypothalamic neurons: Rescue of resistance and apoptosis through adenosine 5' monophosphate-activated protein kinase activation. Endocrinology.

[16] Ulloth J.E., Casiano C.A., De Leon M. (2003). Palmitic and stearic fatty acids induce caspase-dependent and-independent cell death in nerve growth factor differentiated PC12 cells. J. Neurochem..

[17] Beydoun M.A., Kaufman J.S., Satia J.A., Rosamond W., Folsom A.R. (2007). Plasma n−3 fatty acids and the risk of cognitive decline in older adults: The Atherosclerosis Risk in Communities Study. Am. J. Clin. Nutr..

[18] Conquer J.A., Tierney M.C., Zecevic J., Bettger W.J., Fisher R.H. (2000). Fatty acid analysis of blood plasma of patients with Alzheimer’s disease, other types of dementia, and cognitive impairment. Lipids.

[19] Laitinen M., Ngandu T., Rovio S., Helkala E.-L., Uusitalo U., Viitanen M., Nissinen A., Tuomilehto J., Soininen H., Kivipelto M. (2006). Fat intake at midlife and risk of dementia and Alzheimer’s disease: A population-based study. Dement. Geriatr. Cogn. Disord..

[20] Baquer N.Z., Hothersall J.S., McLean P. (1988). Function and regulation of the pentose phosphate pathway in brain. Curr. Top. Cell. Regul..

[21] Hamilton J.A., Brunaldi K. (2007). A model for fatty acid transport into the brain. J. Mol. Neurosci..

[22] Patil S., Chan C. (2005). Palmitic and stearic fatty acids induce Alzheimer-like hyperphosphorylation of tau in primary rat cortical neurons. Neurosci. Lett..

[23] Patil S., Sheng L., Masserang A., Chan C. (2006). Palmitic acid-treated astrocytes induce BACE1 upregulation and accumulation of C-terminal fragment of APP in primary cortical neurons. Neurosci. Lett..

[24] Maedler K., Spinas G., Dyntar D., Moritz W., Kaiser N., Donath M.Y. (2001). Distinct effects of saturated and monounsaturated fatty acids on β-cell turnover and function. Diabetes.

[25] Umegaki H. (2014). Type 2 diabetes as a risk factor for cognitive impairment: Current insights. Clin. Interv. Aging.

[26] Haan M.N. (2006). Therapy Insight: Type 2 diabetes mellitus and the risk of late-onset Alzheimer’s disease. Nat. Clin. Pract. Neurol..

[27] Desai G., Zheng C., Geetha T., Mathews S.T., White B.D., Huggins K.W., Zizza C.A., Broderick T.L., Babu J.R. (2014). The Pancreas-Brain Axis: Insight into Disrupted Mechanisms Associating Type 2 Diabetes and Alzheimer’s Disease. J. Alzheimers Dis..

[28] De Felice F.G., Ferreira S.T. (2014). Inflammation, defective insulin signaling, and mitochondrial dysfunction as common molecular denominators connecting type 2 diabetes to Alzheimer disease. Diabetes.

[29] Morris J.K., Vidoni E.D., Honea R.A., Burns J.M. (2014). Impaired glycemia increases disease progression in mild cognitive impairment. Neurobiol. Aging.

[30] Yang H.T., Sheen Y.J., Kao C.D., Chang C.A., Hu Y.C., Lin J.L. (2013). Association between the characteristics of metabolic syndrome and Alzheimer’s disease. Metab. Brain Dis..

[31] Stephan B., Wells J., Brayne C., Albanese E., Siervo M. (2010). Increased fructose intake as a risk factor for dementia. J. Gerontol. A Biol. Sci. Med. Sci..

[32] Lakhan S.E., Kirchgessner A. (2013). The emerging role of dietary fructose in obesity and cognitive decline. Nutr. J..

[33] Clore J.N., Allred J., White D., Li J., Stillman J. (2002). The role of plasma fatty acid composition in endogenous glucose production in patients with type 2 diabetes mellitus. Metabolism.

[34] Greenwood C.E., Winocur G. (2005). High-fat diets, insulin resistance and declining cognitive function. Neurobiol. Aging.

[35] Craft S., Dagogo-Jack S.E., Wiethop B.V., Murphy C., Nevins R.T., Fleischman S., Rice V., Newcomer J.W., Cryer P.E. (1993). Effects of hyperglycemia on memory and hormone levels in dementia of the Alzheimer type: A longitudinal study. Behav. Neurosci..

[36] Luchsinger J.A., Reitz C., Patel B., Tang M.-X., Manly J.J., Mayeux R. (2007). Relation of diabetes to mild cognitive impairment. Arch. Neurol..

[37] Rosendorff C., Beeri M.S., Silverman J.M. (2007). Cardiovascular risk factors for Alzheimer’s disease. Am. J. Geriatr. Cardiol..

[38] Kroner Z. (2009). The relationship between Alzheimer’s disease and diabetes: Type 3 diabetes?. Altern. Med. Rev..

[39] Arvanitakis Z., Wilson R.S., Bienias J.L., Evans D.A., Bennett D.A. (2004). Diabetes mellitus and risk of Alzheimer disease and decline in cognitive function. Arch. Neurol..

[40] Cukierman T., Gerstein H., Williamson J. (2005). Cognitive decline and dementia in diabetes—systematic overview of prospective observational studies. Diabetologia.

[41] MacKnight C., Rockwood K., Awalt E., McDowell I. (2002). Diabetes mellitus and the risk of dementia, Alzheimer’s disease and vascular cognitive impairment in the Canadian Study of Health and Aging. Dement. Geriatr. Cogn. Dis..

[42] Becker E.B., Bonni A. (2004). Cell cycle regulation of neuronal apoptosis in development and disease. Prog. Neurobiol..

[43] Wang W., Bu B., Xie M., Zhang M., Yu Z., Tao D. (2009). Neural cell cycle dysregulation and central nervous system diseases. Prog. Neurobiol..

[44] Chenn A., Walsh C.A. (2002). Regulation of cerebral cortical size by control of cell cycle exit in neural precursors. Science.

[45] Karaskov E., Scott C., Zhang L., Teodoro T., Ravazzola M., Volchuk A. (2006). Chronic palmitate but not oleate exposure induces endoplasmic reticulum stress, which may contribute to INS-1 pancreatic β-cell apoptosis. Endocrinology.

[46] Busch A.K., Cordery D., Denyer G.S., Biden T.J. (2002). Expression profiling of palmitate-and oleate-regulated genes provides novel insights into the effects of chronic lipid exposure on pancreatic β-cell function. Diabetes.

[47] Pfaffenbach K.T., Gentile C.L., Nivala A.M., Wang D., Wei Y., Pagliassotti M.J. (2010). Linking endoplasmic reticulum stress to cell death in hepatocytes: Roles of C/EBP homologous protein and chemical chaperones in palmitate-mediated cell death. Am. J. Physiol. Endocrinol. Metab..

[48] Alencar R.C., Cobas R.A., Gomes M.B. (2010). Assessment of cognitive status in patients with type 2 diabetes through the Mini-Mental Status Examination: A cross-sectional study. Diabetol. Metab. Syndr..

[49] Kang R., Wan J., Arstikaitis P., Takahashi H., Huang K., Bailey A.O., Thompson J.X., Roth A.F., Drisdel R.C., Mastro R. (2008). Neural palmitoyl-proteomics reveals dynamic synaptic palmitoylation. Nature.

[50] Cao J., Dai D.-L., Yao L., Yu H.-H., Ning B., Zhang Q., Chen J., Cheng W.-H., Shen W., Yang Z.-X. (2012). Saturated fatty acid induction of endoplasmic reticulum stress and apoptosis in human liver cells via the PERK/ATF4/CHOP signaling pathway. Mol. Cell. Biochem..

[51] Nivala A.M., Reese L., Frye M., Gentile C.L., Pagliassotti M.J. (2013). Fatty acid-mediated endoplasmic reticulum stress *in vivo*: Differential response to the infusion of Soybean and Lard Oil in rats. Metabolism.

[52] Hseu Y.-C., Lee M.-S., Wu C.-R., Cho H.-J., Lin K.-Y., Lai G.-H., Wang S.-Y., Kuo Y.-H., Senthil Kumar K., Yang H.-L. (2012). The chalcone flavokawain B induces G2/M cell-cycle arrest and apoptosis in human oral carcinoma HSC-3 cells through the intracellular ROS generation and downregulation of the Akt/p38 MAPK signaling pathway. J. Agric. Food Chem..

[53] Barboule N., Lafon C., Chadebech P., Vidal S., Valette A. (1999). Involvement of p21 in the PKC-induced regulation of the G2/M cell cycle transition. FEBS Lett..

[54] Raab M.S., Breitkreutz I., Tonon G., Zhang J., Hayden P.J., Nguyen T., Fruehauf J.H., Lin B.K., Chauhan D., Hideshima T. (2009). Targeting PKC: A novel role for beta-catenin in ER stress and apoptotic signaling. Blood.

[55] Shih S.-C., Mullen A., Abrams K., Mukhopadhyay D., Claffey K.P. (1999). Role of protein kinase C isoforms in phorbol ester-induced vascular endothelial growth factor expression in human glioblastoma cells. J. Biol. Chem..

[56] Cherubini A., Andres-Lacueva C., Martin A., Lauretani F., Di Iorio A., Bartali B., Corsi A., Bandinelli S., Mattson M.P., Ferrucci L. (2007). Low plasma N-3 fatty acids and dementia in older persons: The InCHIANTI study. J. Gerontol. A Biol. Sci. Med. Sci..

[57] Morris M.C., Evans D.A., Tangney C.C., Bienias J.L., Schneider J.A., Wilson R.S., Scherr P.A. (2006). Dietary copper and high saturated and trans fat intakes associated with cognitive decline. Arch. Neurol..

[58] Eskelinen M.H., Ngandu T., Helkala E.L., Tuomilehto J., Nissinen A., Soininen H., Kivipelto M. (2008). Fat intake at midlife and cognitive impairment later in life: A population-based CAIDE study. Int. J. Geriatr. Psychiatr..

[59] Morris M., Evans D., Bienias J., Tangney C., Wilson R. (2004). Dietary fat intake and 6-year cognitive change in an older biracial community population. Neurology.

[60] Eitel K., Staiger H., Brendel M.D., Brandhorst D., Bretzel R.G., Häring H.-U., Kellerer M. (2002). Different role of saturated and unsaturated fatty acids in β-cell apoptosis. Biochem. Biophys. Res. Commun..

[61] Montell E., Turini M., Marotta M., Roberts M., Noé V., Macé K., Gómez-Foix A.M. (2001). DAG accumulation from saturated fatty acids desensitizes insulin stimulation of glucose uptake in muscle cells. Am. J. Physiol. Endocrinol. Metab..

[62] Listenberger L.L., Ory D.S., Schaffer J.E. (2001). Palmitate-induced apoptosis can occur through a ceramide-independent pathway. J. Biol. Chem..

[63] Staiger K., Staiger H., Weigert C., Haas C., Häring H.-U., Kellerer M. (2006). Saturated, but not unsaturated, fatty acids induce apoptosis of human coronary artery endothelial cells via nuclear factor-κB activation. Diabetes.

[64] Mu Y.-M., Yanase T., Nishi Y., Tanaka A., Saito M., Jin C.-H., Mukasa C., Okabe T., Nomura M., Goto K. (2001). Saturated FFAs, palmitic acid and stearic acid, induce apoptosis in human granulosa cells. Endocrinology.

[65] Trombetta A., Togliatto G., Rosso A., Dentelli P., Olgasi C., Cotogni P., Brizzi M.F. (2013). Increase of palmitic acid concentration impairs endothelial progenitor cell and bone marrow-derived progenitor cell bioavailability: Role of the STAT5/PPARgamma transcriptional complex. Diabetes.

[66] Yuan Q., Zhao S., Wang F., Zhang H., Chen Z.J., Wang J., Wang Z., Du Z., Ling E.A., Liu Q., Hao A. (2013). Palmitic acid increases apoptosis of neural stem cells via activating c-Jun N-terminal kinase. Stem Cell Res..

[67] Pereira D.M., Correia-da-Silva G., Valentão P., Teixeira N., Andrade P.B. (2013). Palmitic Acid and Ergosta-7, 22-dien-3-ol Contribute to the Apoptotic Effect and Cell Cycle Arrest of an Extract from Marthasterias glacialis L. in Neuroblastoma Cells. Mar. Drugs.

[68] Singh S.V., Herman-Antosiewicz A., Singh A.V., Lew K.L., Srivastava S.K., Kamath R., Brown K.D., Zhang L., Baskaran R. (2004). Sulforaphane-induced G2/M phase cell cycle arrest involves checkpoint kinase 2-mediated phosphorylation of cell division cycle 25C. J. Biol. Chem..

[69] Teodoro J.G., Heilman D.W., Parker A.E., Green M.R. (2004). The viral protein Apoptin associates with the anaphase-promoting complex to induce G2/M arrest and apoptosis in the absence of p53. Genes Dev..

[70] Bourougaa K., Naski N., Boularan C., Mlynarczyk C., Candeias M.M., Marullo S., Fåhraeus R. (2010). Endoplasmic reticulum stress induces G2 cell-cycle arrest via mRNA translation of the p53 isoform p53/47. Mol. Cell.

[71] Patil S., Melrose J., Chan C. (2007). Involvement of astroglial ceramide in palmitic acid-induced Alzheimer-like changes in primary neurons. Eur. J. Neurosci..

[72] Yehuda S., Rabinovitz S., Carasso R.L., Mostofsky D.I. (2002). The role of polyunsaturated fatty acids in restoring the aging neuronal membrane. Neurobiol. Aging.

[73] Wei Y., Wang D., Pagliassotti M.J. (2007). Saturated fatty acid-mediated endoplasmic reticulum stress and apoptosis are augmented by trans-10, cis-12-conjugated linoleic acid in liver cells. Mol. Cell. Biochem..

[74] Madaro L., Marrocco V., Carnio S., Sandri M., Bouché M. (2013). Intracellular signaling in ER stress-induced autophagy in skeletal muscle cells. FASEB J..

[75] Larroque-Cardoso P., Swiader A., Ingueneau C., Nègre-Salvayre A., Elbaz M., Reyland M., Salvayre R., Vindis C. (2013). Role of protein kinase C δ in ER stress and apoptosis induced by oxidized LDL in human vascular smooth muscle cells. Cell Death Dis..

[76] Benoit S.C., Kemp C.J., Elias C.F., Abplanalp W., Herman J.P., Migrenne S., Lefevre A.L., Cruciani-Guglielmacci C., Magnan C., Yu F. (2009). Palmitic acid mediates hypothalamic insulin resistance by altering PKC-theta subcellular localization in rodents. J. Clin. Invest..

[77] Voss O.H., Kim S., Wewers M.D., Doseff A.I. (2005). Regulation of monocyte apoptosis by the protein kinase Cdelta-dependent phosphorylation of caspase-3. J. Biol. Chem..

[78] Rocks O., Gerauer M., Vartak N., Koch S., Huang Z.P., Pechlivanis M., Kuhlmann J., Brunsveld L., Chandra A., Ellinger B. (2010). The palmitoylation machinery is a spatially organizing system for peripheral membrane proteins. Cell.

[79] Greaves J., Prescott G.R., Gorleku O.A., Chamberlain L.H. (2009). The fat controller: Roles of palmitoylation in intracellular protein trafficking and targeting to membrane microdomains (Review). Mol. Membr. Biol..

[80] Salaun C., Greaves J., Chamberlain L.H. (2010). The intracellular dynamic of protein palmitoylation. J. Cell Biol..

[81] Fukata Y., Fukata M. (2010). Protein palmitoylation in neuronal development and synaptic plasticity. Nat. Rev. Neurosci..

[82] Bijlmakers M.-J., Marsh M. (2003). The on–off story of protein palmitoylation. Trends Cell Biol..

[83] Salminen A., Kauppinen A., Suuronen T., Kaarniranta K., Ojala J. (2009). ER stress in Alzheimer’s disease: A novel neuronal trigger for inflammation and Alzheimer’s pathology. J. Neuroinflammation.

[84] Baldwin A.C., Green C.D., Olson L.K., Moxley M.A., Corbett J.A. (2012). A role for aberrant protein palmitoylation in FFA-induced ER stress and β-cell death. Am. J. Physiol. Endocrinol. Metab..

[85] Borradaile N.M., Han X., Harp J.D., Gale S.E., Ory D.S., Schaffer J.E. (2006). Disruption of endoplasmic reticulum structure and integrity in lipotoxic cell death. J. Lipid Res..

[86] Resh M.D. (2006). Use of analogs and inhibitors to study the functional significance of protein palmitoylation. Methods.

[87] Webb Y., Hermida-Matsumoto L., Resh M.D. (2000). Inhibition of protein palmitoylation, raft localization, and T cell signaling by 2-bromopalmitate and polyunsaturated fatty acids. J. Biol. Chem..

[88] Smotrys J.E., Linder M.E. (2004). Palmitoylation of intracellular signaling proteins: Regulation and function. Ann. Rev. Biochem..

[89] Uhrig M., Ittrich C., Wiedmann V., Knyazev Y., Weninger A., Riemenschneider M., Hartmann T. (2009). New Alzheimer amyloid beta responsive genes identified in human neuroblastoma cells by hierarchical clustering. PLoS One.

[90] Hamid R., Rotshteyn Y., Rabadi L., Parikh R., Bullock P. (2004). Comparison of alamar blue and MTT assays for high through-put screening. Toxicol In Vitro.

[91] Folch J., Lees M., Sloane-Stanley G. (1957). A simple method for the isolation and purification of total lipids from animal tissues. J. Biol. Chem..

[92] Dobrzyń A., Górski J. (2002). Ceramides and sphingomyelins in skeletal muscles of the rat: Content and composition. Effect of prolonged exercise. Am. J. Physiol. Endocrinol. Metab..

[93] Tserng K.-Y., Griffin R. (2003). Quantitation and molecular species determination of diacylglycerols, phosphatidylcholines, ceramides, and sphingomyelins with gas chromatography. Anal. Biochem..

[94] Darzynkiewicz Z., Juan G. (2001). DNA content measurement for DNA ploidy and cell cycle analysis. Curr. Protoc. Cytom..

[95] Lee K.-T., Chen Y.-H., Lin C.-I., Chiu W.-C., Liao H., Lin S.-H. (2013). Consumption of oriental plums improved the cognitive performance and modulated the cerebral neurodegeneration-related protein expressions in rats with nicotinamide/streptozotocin-induced diabetes. Food Nutr. Sci..

[96] Westermann P., Knoblich M., Maier O., Lindschau C., Haller H. (1996). Protein kinase C bound to the Golgi apparatus supports the formation of constitutive transport vesicles. Biochem. J..

[97] Bruns R.F., Miller F.D., Merriman R.L., Howbert J.J., Heath W.F., Kobayashi E., Takahashi I., Tamaoki T., Nakano H. (1991). Inhibition of protein kinase C by calphostin C is light-dependent. Biochem. Biophys. Res. Commun..

